# Basal-like grade III invasive ductal carcinoma of the breast: patterns of metastasis and long-term survival

**DOI:** 10.1186/bcr1636

**Published:** 2007-01-11

**Authors:** Laura G Fulford, Jorge S Reis-Filho, Ken Ryder, Chris Jones, Cheryl E Gillett, Andrew Hanby, Douglas Easton, Sunil R Lakhani

**Affiliations:** 1 Breakthrough Breast Cancer Research Centre, The Institute of Cancer Research, Fulham Road, London, SW3 6JB, UK; 2 The Ludwig Institute for Cancer Research, 91 Riding House Street, London W1W 7BS, UK; 3 Hedley Atkins/Imperial Cancer Research Fund Breast Pathology Laboratory, Guy's Hospital, London, SE1 9RT, UK; 4 The Institute of Cancer Research, Fulham Road, London, SW3 6JB, UK; 5 Academic Unit of Pathology, Leeds University c/o St James' University Hospital, Beckett Street, Leeds, LS9 7TF, UK; 6 Cancer Research Campaign Genetic Epidemiology Unit, Strangeways Research Laboratory, Worts Causeway, Cambridge, CB1 8RN, UK; 7 Molecular and Cellular Pathology, School of Medicine, The University of Queensland, and Queensland Institute of Medical Research, 300 Herston Road, Herston, Brisbane, 4006, Australia

## Abstract

**Introduction:**

Cytokeratin (CK) 14, one of several markers expressed in normal myoepithelial/basal cells, is also expressed in a proportion of breast carcinomas. Previous studies have suggested that expression of such 'basal' markers predicts different biological behaviour, with more frequent lung and brain metastases and poorer prognosis than other carcinomas.

**Methods:**

We performed CK14 immunohistochemistry on 443 grade III invasive ductal carcinomas with extended clinical follow-up (mean 116 months), and we correlated CK14 immunopositivity (basal-like phenotype) with clinicopathological criteria.

**Results:**

Eighty-eight of 443 (20%) tumours showed CK14 expression. CK14-positive tumours were more likely to be oestrogen receptor-negative (*p *< 0.0001) and axillary node-negative (*p *= 0.001) than were CK14-negative cases. CK14-positive cases developed less bone and liver metastases (hazard ratio [HR] 0.49, *p *= 0.01, and HR 0.53, *p *= 0.035, respectively) but more frequent brain metastases (HR 1.92, *p *= 0.051). In patients without metastatic disease, disease-free survival in CK14-positive cases was significantly better than in CK14-negative cases (HR 0.65, *p *= 0.005). In patients with metastatic disease, however, CK14 positivity was associated with a poorer prognosis (HR 1.84, *p *= 0.001). The overall survival in CK14-positive and -negative patients was similar at 5 years (60% and 59%, respectively), but the long-term survival was better in CK14-positive patients (HR 0.69, *p *= 0.02).

**Conclusion:**

These results demonstrate that basal-like tumours differ in their biological behaviour from other tumours, with a distinct pattern of metastatic spread. Compared to other grade III tumours, basal-like tumours appear to have a relatively good long-term survival but survival after metastases is poor.

## Introduction

Breast cancer is a common but very diverse disease with considerable survival heterogeneity [1-5]. An ongoing challenge is to find improved methods of identifying and classifying groups of tumours with differing biological behaviours or responsiveness to specific therapies. Expression profiling analysis is reshaping our understanding of breast cancer and has provided a working model for modern breast cancer taxonomy [6-8]. These seminal studies have systematically identified a subgroup of breast tumours that are characterised by the expression of genes normally expressed in basal/myoepithelial cells of normal breast, including high-molecular-weight cytokeratins (CKs) and P-cadherin [6-10]. These findings have confirmed and expanded the concepts first described as early as 1967 [11], and in particular those described over the last decade [12], by conventional histopathological and immunohistochemical examination. Studies have demonstrated that whilst most breast tumours express a purely luminal phenotype, a minority of ductal tumours (4% to 22%) will show ultrastructural features and co-express markers that are characteristic of the outer myoepithelial, or basal, compartment of the normal breast [11-35].

**Figure 1:**
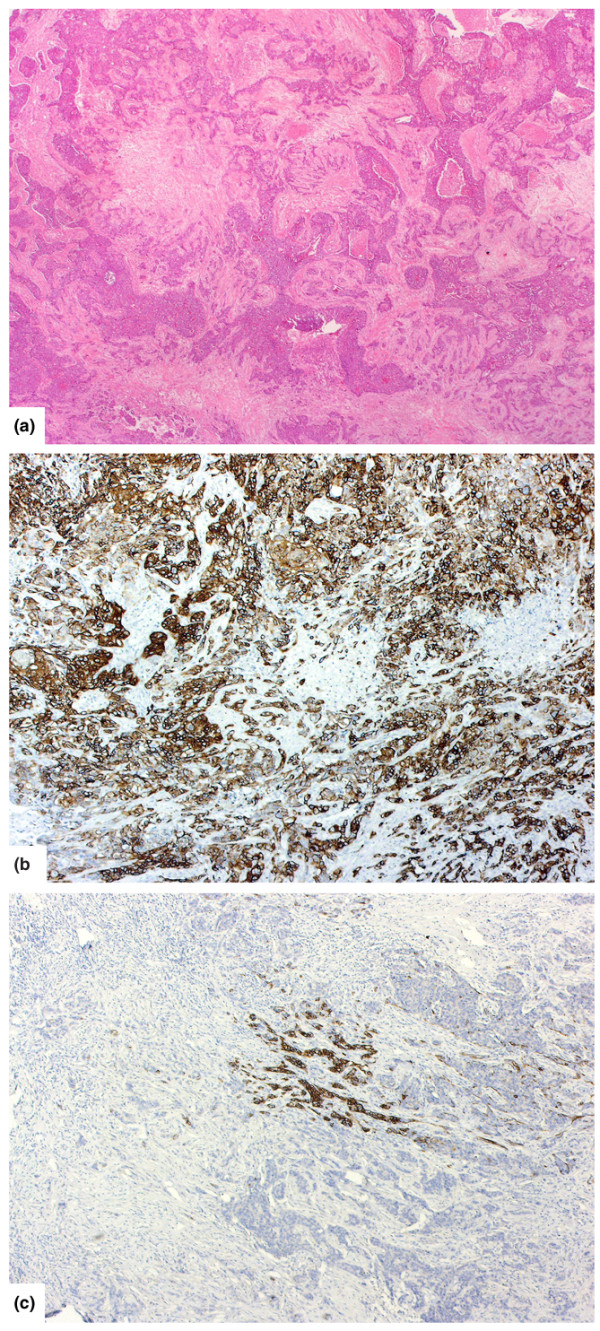
Histological appearance of basal tumours and CK14 expression patterns. **(a) **Typical histological appearance of a basal breast carcinoma (haematoxylin and eosin). **(b) **CK14 expression: a basal tumour with basal keratin expression in almost every tumour cell (diffuse staining pattern). **(c) **Another basal tumour with only a minority of tumour cells showing expression (focal staining pattern).

Morphologically, these tumours are predominantly of high grade [9,19,23-26,29,35] and more frequently show medullary-like features [30,31] and metaplastic elements [19,20,32,33,36-39] (Figure 1a). Immunohistochemically, they are predominantly oestrogen receptor (ER)-, progesterone receptor (PR)-, and C-erb B2 receptor (HER2)-negative [19,22,24,25,32]. These characteristics also predominate among tumours arising in patients with germline *BRCA1 *(breast cancer 1, early onset) mutations [40,41]. Recent studies have indicated that positive staining for 'basal' markers in patients with familial breast and/or ovarian tumours is strongly predictive of carrying a *BRCA1 *mutation [27,34,41]; however, there are several lines of evidence to suggest that sporadic basal-like ductal carcinomas do not harbour *BRCA1 *somatic mutations [42].

Despite these morphological, immunohistochemical, and molecular features, current routine diagnostic practice does not separately recognise these tumours and management is the same as for other grade- and stage-matched breast tumours. Importantly, however, evidence suggests that these tumours have a metastatic pattern of dissemination and clinical behaviour that are different than comparable ductal carcinomas not showing basal marker expression. Tsuda *et al*. [19] and Hicks *et al*. [43] reported a higher incidence of lung and brain metastases associated with a myoepithelial phenotype, as defined by CK14, smooth muscle actin, or S100 protein expression. Furthermore, retrospective, population-based studies have demonstrated that tumours that express these basal markers have a more aggressive clinical behaviour when compared to those with a luminal phenotype and that 'basal' status may be an independent prognostic factor. In contrast, a comparative genomic hybridisation (CGH) analysis performed in our institutions [28] of 86 invasive ductal carcinomas of no special type (IDC-NST) (50% CK14-positive and stage- and age-matched), all of which were grade III, did not suggest a uniformly poor prognosis for the basal-like (CK14-positive) tumours. Instead, the CGH analysis revealed distinct subgroups of basal-like grade III tumours that exhibited a comparatively good prognosis with extended clinical follow-up (>10 years), in comparison to the rest of the grade III group, and a basal-like subgroup with a relatively poor prognosis. In fact, in contrast to the 'pan-grade' studies, the CK14-positive tumours appeared to have an overall better outcome than the rest of the group [28].

To investigate these observations further and explore the hypothesis that basal-like tumours are entities biologically distinct from other ductal carcinomas and that they may themselves have different subgroups, we assessed a large cohort of grade III IDC-NST for expression of CK14 and correlated this with long-term survival and pattern of metastatic disease.

## Materials and methods

Four hundred and seventy grade III IDC-NST, diagnosed between 1975 and 1991, were identified from the patient database at the Hedley Atkins/Imperial Cancer Research Fund Breast Pathology Laboratory, Guy's Hospital (London, UK), where all the cases were managed. Tumours had been graded according to the modified Bloom and Richardson system [2] and have been previously published [44]. Immunohistochemistry was performed on a single tumour block for CK14 as detailed previously [28,29]. Briefly, 2 minutes of pressure-cooking at pH 6.0 was used for antigen retrieval with CK14 clone LL002 (1:50) (BioGenex Inc., San Ramon, CA, USA). Four hundred and forty-three cases contained tumour on review.

CK14 expression was assessed independently by two pathologists (LGF and JSR-F). A tumour was designated as CK14-positive if it was considered that at least 1% of true invasive tumour cells expressed the CK marker. From our previous experience [28,29], and as recently demonstrated by Laakso *et al*. [25,26], it has been apparent that whereas in some tumours virtually every cell expresses the basal marker (CK14, in this instance), others may show a focal or variable percentage of positive cells (Figure 1b,c). We therefore subdivided the CK14-positive tumours into those showing 'diffuse expression' (defined as more than 90% of tumour cells positive; Figure 1b) and those showing 'focal expression' (1% to 90% of cells positive; Figure 1c). Discrepancies between the observers were resolved on a multi-headed microscope. For ease of reading, CK14-positive tumours are referred to henceforth as 'basal' and CK14-negative tumours as 'non-basal' cases. Data on tumour size, axillary lymph node status, ER, and primary treatment were available for 98%, 98%, 93%, and 100% of patients, respectively. Between 1975 and 1991, the methodology for ER measurement changed as newer reagents became available. Initially, ER status was determined by cytosol ligand-binding assay [45], which was then replaced by the Abbott enzyme immunoassay (EIA) (Abbott Laboratories, Abbott Park, IL, USA) [46]. A successful comparative study was undertaken between the ligand-binding assay and EIA before transferring to the latter method.

All ER data were recorded on a prospectively acquired and verified clinicopathological database at the time of assay. Full clinical follow-up data, including sites of metastatic disease, were available for all cases.

### Statistical analysis

Associations between CK14 immunoreactivity and clinicopathological parameters–including tumour size (as defined by TMN [tumour-node-metastasis] staging, 5^th ^edition), tumour grade (modified Bloom-Richardson grading) [2], menstrual status, ER and PR status, presence of axillary lymph node metastases, local recurrence, and distant metastasis–were evaluated by Fisher's exact test or χ^2 ^test as appropriate. Mean age at diagnosis and CK14 positivity were compared using a *t *test.

Survival analyses were conducted for overall survival (OS), disease-free survival (DFS), survival to metastases at specific sites, and time from first recurrence to death. DFS was defined as time to any type of recurrence, distant metastasis, or death from any cause. Survival curves were calculated using the Kaplan-Meier method. Tests of differences in survival between groups were performed using the log-rank test and were expressed as hazard ratios (HRs), which were estimated using Cox regression. Cox regression analysis was also used to evaluate any independent effect of prognostic factors on DFS, OS, and survival from recurrence. Factors significant at a *p *value of less than 0.05 in the survival analysis, together with CK14 as the factor of principal interest, were included in the regression analyses. All tests were two-tailed, and 95% confidence intervals are presented where appropriate. All analyses were carried out using Stata (version 7.0, StataCorp LP, Texas, USA).

## Results

The distribution of clinicopathological features is detailed in Table 1. Briefly, age at diagnosis ranged from 21 to 85 years (mean 53.2 years). Fifty-seven percent of tumours had axillary nodal metastases, and 46% were ER-positive. Fifty-two percent of cases had received no form of adjuvant treatment. Length of follow-up ranged from 3.2 to 333.5 months, with a mean follow-up of 116.4 months.

### OS and DFS in the whole cohort

Associations of potential prognostic factors with OS, DFS, and survival from first recurrence are shown in Table 2. As expected, both OS and recurrence-free survival were inversely related to the presence of axillary nodal metastases (*p *< 0.0001) and primary tumour size (*p *= 0.003). Tumours occurring in women less than 35 years old or more than 65 years old at diagnosis had poorer OS and DFS than women 35 to 65 years old (*p *= 0.002 and *p *= 0.01, respectively). ER- and PR-negative tumours showed a significantly poorer survival from first recurrence (HR 2.10, *p *< 0.0001, and HR 1.51, *p *= 0.005, respectively). These features are entirely consistent with the outcomes expected in follow-up of such a group of grade III breast tumours.

### CK14 expression

Of the 443 cases, 88 (20%) showed CK14 expression and are considered to be 'basal' tumours. They occurred at a slightly, but significantly, younger age than other grade III tumours (mean 49.9 years versus 53.9 years, *p *= 0.0065; Table 3). They were more likely to be ER- and PR-negative (odds ratio [OR] 10.9, *p *< 0.0001, and OR 11.9, *p *< 0.0001, respectively) and less likely to have axillary nodal metastases (OR 0.44, *p *= 0.0023). There was no apparent correlation with tumour size or menopausal status.

### Basal phenotype and effect on survival

**Figure 2:**
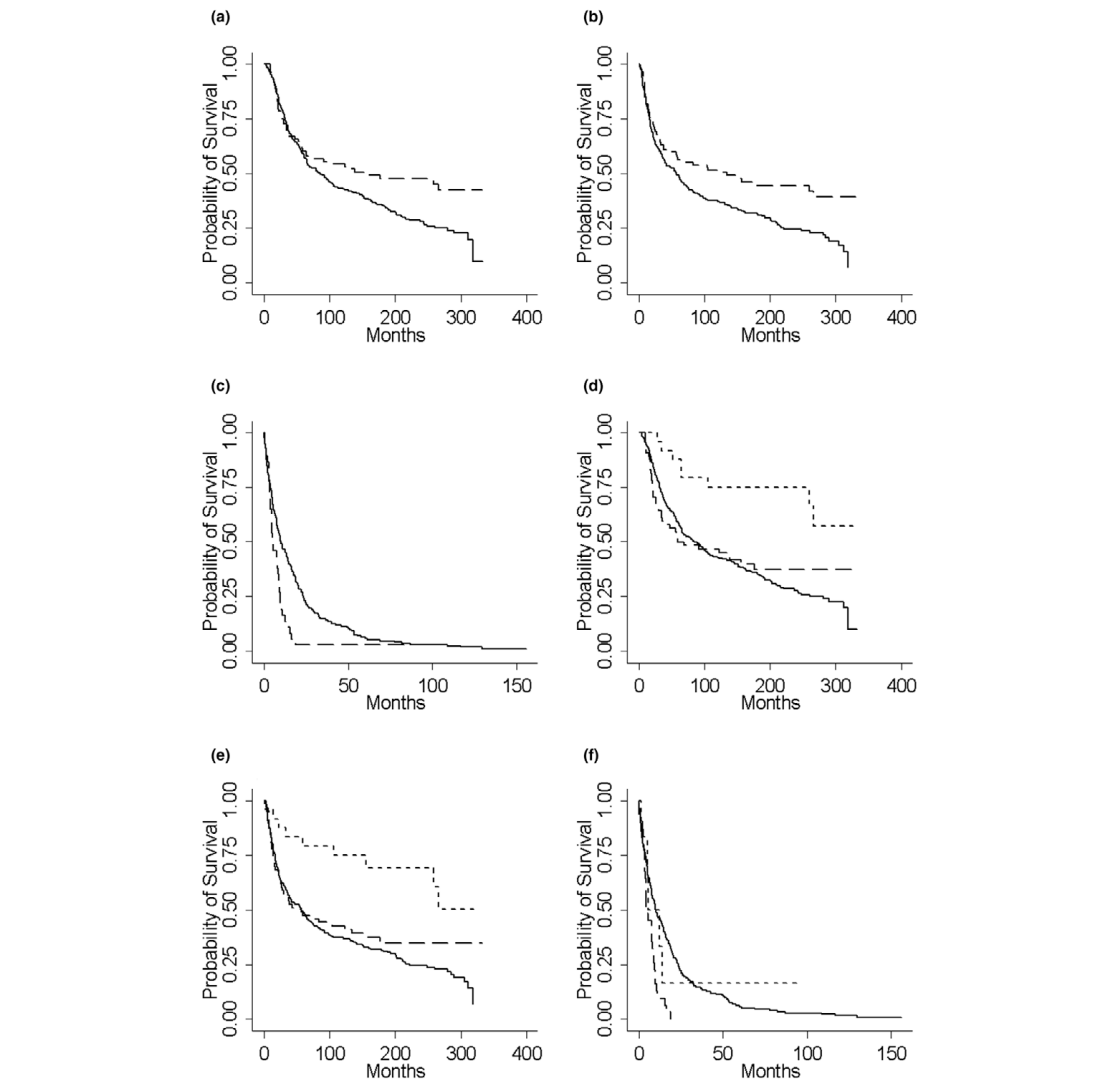
Long term survival in basal versus non-basal tumours. **(a-c) **Kaplan-Meier survival plots for basal (dashed line) versus non-basal (solid line) tumours. **(a) **Overall survival, **(b) **disease-free survival, and **(c) **survival from metastasis. **(d-f) **Kaplan-Meier survival plots for diffuse basal (short dashes) versus focal basal (long dashes) versus non-basal (solid line) tumours. **(d) **Overall survival, **(e) **disease-free survival, and **(f) **survival from metastasis.

Basal tumours exhibited a significantly better OS and DFS than non-basal tumours (HR 0.69, *p *= 0.02, and HR 0.65, *p *= 0.005, respectively; Table 2, Figure 2a,b). DFS was similar in basal and non-basal patients for the first 5 years after diagnosis (56% in basal patients, 49% in non-basal patients) but then diverged (52% versus 38% at 10 years). Similarly, the OS was almost identical during the first 5 years (60% versus 59%) but then diverged such that patients with basal tumours survived significantly longer (survival at 10 years 54% versus 43%). The difference in survival remained when analyses were restricted to ER-negative tumours (HR 0.62, *p *= 0.01, for OS; HR 0.63, *p *= 0.01, for DFS). After adjustment for age, tumour size, and nodal metastases, DFS remained significantly better in basal cases (HR 0.72, *p *= 0.05) but the effect on OS was no longer significant (HR 0.78, *p *= 0.14; Table 4).

Importantly, considering only breast tumours that relapsed, expression of basal markers was associated with significantly poorer prognosis. Survival after first recurrence was significantly shorter for basal tumours (HR 1.84, *p *= 0.001; Figure 2c). Interestingly, most of the recurrences seen in patients with basal tumours happened in the first 5 years of follow-up.

### CK14 expression patterns

Of the 88 basal cases, 24 (27%) fell into the diffuse staining category and the remaining 64 (73%) showed focal CK14 expression (of these, half exhibited less than 50% tumour cell expression and the remainder between 50% and 90%). Both OS and DFS were significantly better for diffuse than for focal CK14 staining (*p *= 0.012 and *p *= 0.02, respectively). Focally positive tumours had OS and DFS similar to the non-basal tumours, whereas the prognosis for diffuse staining was markedly better (Figure 2d,e). Survival from metastasis did not differ by staining pattern (*p *= 0.14), but this comparison had very limited power because there were only nine recurrences in the diffuse staining group (Figure 2f). Statistical analysis showed no difference in hormone status, tumour size, axillary nodal status, menopausal status, age, or local recurrence between tumours showing the two different patterns of staining (data not shown).

### Effect of treatment

Because these tumours date from the 1970s, there was wide variation in treatments. Some patients received tamoxifen and/or chemotherapy, whereas the majority received no systemic therapy, as was standard at that time. To investigate whether the differences in survival seen in the basal tumours may be related to the introduction of adjuvant therapies, we also analysed the subset of 229 patients who had received no adjuvant chemotherapy, hormonal therapy, or ovarian oblation. In this subset, basal tumours (*n *= 52, 23%) were still associated with a significantly improved OS (HR 0.50, *p *= 0.018) and DFS (HR 0.48, *p *= 0.001); however, this effect was not independent of the nodal status, because basal tumours are more often node-negative at presentation. These HRs were greater than for the cohort as a whole (tests for interaction, *p *= 0.01 for OS and *p *= 0.017 for DFS). Among patients treated with adjuvant hormonal therapy, basal tumours were associated with a poorer survival (HR 1.76, 95% confidence interval 0.94 to 3.29). This is likely to reflect the inverse correlation between expression of basal markers and hormone receptors and the impact of hormone therapy on the survival of patients with receptor-positive breast cancer.

### Basal tumours and sites of metastases

There were clear differences in the development of metastases to specific sites between the basal and non-basal tumours (Table 5). Basal tumours were significantly less likely to develop bone (*p *= 0.01) or liver (*p *= 0.035) metastases or have involvement of non-regional lymph nodes (*p *= 0.008) but were more likely to develop brain metastases (*p *= 0.051). There was no difference in the rate of lung and pleural metastases between the two groups.

## Discussion

These data contrast with much of the recently published data showing entirely poor outcome in basal tumours [7,8,10,17,33]. Unlike that of previous pan-grade studies, the aim of this study was to look at the impact of 'basal' phenotype on the long-term outcome in patients with grade III IDC-NST, a subgroup with a traditionally poor prognosis. We have specifically chosen a cohort of cases diagnosed historically as grade III ductal carcinomas, not including any special subtypes such as metaplastic breast carcinoma or medullary carcinoma, because this is the group of tumours in which the majority of basal cases seem to arise [5].

Terminology and definitions surrounding the concept of basal tumours are controversial, and a plethora of different markers and definitions have been employed to identify cases in clinical studies. Nielsen and colleagues [22] have proposed a definition based on ER, HER2, CK5/6, and EGFR (epidermal growth factor receptor) expression; however, even this definition does not show a perfect correlation with microarray data. Indeed, there is no accepted gold standard on which to assess any signature (for example, in a recent study [9], only 80% of tumours classified as HER2 by cDNA microarrays overexpressed HER2 at the immunohistochemical level). Given that there is currently no consensus terminology or internationally accepted definition (morphological or immunophenotypic) for basal-like tumours, we have employed CK14 alone to identify basal tumours in the present study. Our reasons for choosing a single marker are pragmatic: (a) limited material from this unique cohort of patients was available, and (b) CK14 is a marker that we have had considerable experience with and forms the basis for previous studies in the laboratory [28,29,36]. Furthermore, it has been demonstrated that CK14 expression correlates strongly with other 'basal' markers and that it identifies basal tumour proportions similar to those of other investigators (for example, CK5/6, CK17, and P-cadherin) [21-26,32,33,35].

Our results have highlighted several important features of basal tumours as identified by CK14 expression. Relative to other grade III tumours, they are much more likely to be ER-negative and less likely to be axillary node-positive. They show lower rates of metastasis to the bone and liver but a higher rate of brain metastasis. Over the first 5 years after diagnosis, the relapse-free survival and OS appear to be similar to other grade III tumours, but their subsequent prognosis is much better. Given that in this study we compared grade III tumours only and that basal-like phenotype and 3+ expression of HER2 are more prevalent in this group but are inversely correlated [9,22,39,47], it is likely that the non-basal-like tumours include a high prevalence of HER2-amplified tumours [47], which are reported to have an aggressive clinical behaviour. Further studies to directly compare the contribution of basal-like and HER2 phenotypes to survival of patients with grade III ductal carcinomas are warranted but are outside the remit of this study. The pattern of recurrence in basal tumours, with almost all recurrences occurring in the first 5 years, also differs from non-basal tumours, in which late relapse is widely recognised. Previous studies, many of which have a shorter median follow-up than that of our cohort of patients, have indicated that basal tumours usually have a very poor prognosis when compared to a mixture of grades I, II, and III basal keratin-negative breast tumours [7,8,10,17,32,33]. These observations are not necessarily inconsistent with this study, because our study does indicate a poor survival for patients with basal tumours for the first 5 years of follow-up.

The findings suggest the existence of two subgroups of basal carcinomas: one exhibiting early relapse and aggressive clinical course and a separate group that despite the traditionally poor prognostic indicators do not relapse. This would be consistent with the findings of our previous CGH analysis [28], which was carried out on a subset of the tumours examined in the present study and was able to split the basal tumours into two groups based on both their molecular genetic alterations and prognosis.

We found that traditional clinicopathological parameters could not discriminate between 'good' and 'bad' basal tumours. In addition, we found that none of the specific morphological features associated with the basal phenotype [36], such as central scar, high mitotic rates, pushing growth pattern, or necrosis, occurred with any significant difference between good and bad basal cases. However, the pattern of CK14 expression shows clear differences in prognosis. Those with a diffuse (>90%; Figure 1b) staining pattern seem to fall largely into the good-prognosis group of tumours. A similar subclassification of basal tumours according to the expression of basal keratins into basal and basoluminal subtypes also suggested that tumours with a pure basal phenotype had a better prognosis [26].

Because the majority of breast tumours in *BRCA1 *germline mutation carriers are of basal type [27,34,41], our observations are also relevant to the prognosis in these women. Studies of prognosis in *BRCA1 *germline mutation carriers have been inconsistent; studies based on carriers identified through clinical testing in high-risk families have shown a relatively good prognosis, whereas studies based on testing of population series have shown a poor prognosis [48]. This apparent inconsistency could be explained by our results because cases undergoing clinical testing will tend to be identified some time after diagnosis when subsequent prognosis is good.

These concepts have important implications for patient management. This study confirms that there is a group of aggressive basal tumours that are currently not identified in routine diagnostic practice but that show a biological behaviour different from other grade III IDC-NST. They relapse early with short subsequent survival and notably carry a higher risk of brain metastases [19,43]. These tumours may need different (for example, platinum-based) [49] chemotherapy regimens or more aggressive therapeutic regimens and may need to be specifically identified in laboratory research designed to identify new therapeutic targets.

It is also clear from these results that expression of basal markers alone cannot be used to indicate a tumour with poor prognosis. Indeed, it may be that some of the basal tumours with a better prognosis will require much less aggressive therapy and follow-up and that patients can be reassured of their long-term outlook despite being diagnosed with a grade III cancer.

## Conclusion

This study shows that identifying basal tumours as a distinct group has real clinical relevance. They are relatively common, occurring in approximately 20% of grade III IDC-NST and approximately 10% of breast tumours overall. In the UK, with approximately 50,000 new breast tumours diagnosed each year, we may be missing some 5,000 basal tumours that carry distinct, but mixed, prognostic implications and that may respond differently to conventional chemotherapy regimens. The aim now is finding ways to further identify, characterise, and define 'good' and 'bad' basal tumours and to tailor management appropriately. Furthermore, these tumours may express distinct targets for novel therapeutic regimens, and research programmes should bear this in mind. Clinicopathological, therapeutic, and molecular genetic approaches will almost certainly all play a part in this task.

## Abbreviations

*BRCA1 *= breast cancer 1, early onset; CGH = comparative genomic hybridisation; CK = cytokeratin; DFS = disease-free survival; EIA = enzyme immunoassay; ER = oestrogen receptor; HER2 = C-erb B2 receptor; HR = hazard ratio; IDC-NST = invasive ductal carcinomas of no special type; OR = odds ratio; OS = overall survival; PR = progesterone receptor.

## Competing interests

The authors declare that they have no competing interests.

## Authors' contributions

LGF conceived the study, analysed the immunohistochemical markers, accrued and collated the data, carried out preliminary statistical analysis, supervised the statistical analysis, and drafted the manuscript. JSR-F analysed the immunohistochemical markers, accrued and collated the data, carried out preliminary statistical analysis, and drafted the manuscript. KR and CEG curated the data. CJ and AH participated in the study design. DE supervised and reviewed the statistical analysis. SRL conceived the study, supervised the statistical analysis, and drafted the manuscript. All authors read and approved the final manuscript.

## Figures and Tables

**Table 1:** Clinicopathological features of the cohort of 470 grade III invasive ductal carcinomas of no special type

Parameter	Number
Age in years	
Range	21–85 (mean 53.2)
<35	36 (8%)
35–50	142 (32%)
51–65	189 (43%)
>65	76 (17%)
Menopausal status	
Premenopausal	175 (40%)
Perimenopausal	64 (15%)
Postmenopausal	195 (45%)
Unknown	9
Tumour size	
Range	0.5–10.5 cm (mean 3.4 cm)
p1	27 (6%)
p2	313 (72%)
p3	93 (21%)
Axillary nodes	
Positive	251 (57%)
Negative	184 (42%)
Oestrogen receptor	
Positive	191 (46%)
Negative	222 (54%)
Not available	30
Surgical management	
None	2 (1%)
Mastectomy	318 (72%)
Tumourectomy	123 (28%)
Adjuvant treatment	
None	229 (52%)
Tamoxifen/Ovarian oblation	103 (22%)
Chemotherapy and tamoxifen	5 (1%)
Chemotherapy	112 (25%)
Length of follow-up in months	3.2–333.5 (mean 116.4)

Tumour sizes: p1, <2 cm; p2, 2–5 cm; p3, >5 cm.

**Table 2:** Overall survival and disease-free survival data for all tumours

Parameter	Overall survival			Disease-free survival			Survival from recurrence	
	
	5-year	Hazard ratio (95% CI)	*p *value	5-year	Hazard ratio (95% CI)	*p *value	Hazard ratio (95% CI)	*p *value
Axillary metastases								
No	71%	1.0	<0.0001	65%	1.0	<0.0001	1.0	0.32
Yes	51%	1.83 (1.44–2.33)		41%	1.83 (1.45–2.32)		1.17 (0.86–1.57)	
Tumour size								
p1	89%	1.0	0.002	70%	1.0	0.0005	1.0	0.26
p2	61%	1.54 (0.92–2.62)		54%	1.49 (0.90–2.48)		1.09 (0.60–1.96)	
p3	46%	2.18 (1.25–3.82)		35%	2.20 (1.28–3.79)		1.29 (0.69–2.41)	
ER								
Positive	64%	1.0	0.68	52%	1.0	0.74	1.0	<0.0001
Negative	52%	1.05 (0.83–1.33)		48%	0.96 (0.76–1.21)		2.1 (1.59–2.79)	
PR								
Positive	64%	1.0	0.82	49%	1.0	0.55	1.0	0.005
Negative	56%	1.03 (0.80–1.33)		52%	0.93 (0.72–1.19)		1.51 (1.13–2.02)	
Age in years								
<35	53%	1.0	0.002	39%	1.0	0.01	1.0	0.96
35–50	58%	0.90 (0.57–1.41)		50%	0.99 (0.63–1.55)		0.89 (0.57–1.39)	
50–65	64%	0.81 (0.52–1.26)		57%	0.85 (0.55–1.31)		0.89 (0.57–1.38)	
>65	54%	1.44 (0.90–2.30)		44%	1.39 (0.87–2.23)		0.89 (0.52–1.50)	
CK14								
Negative	59%	1.0	0.02	49%	1.0	0.005	1.0	0.001
Positive	60%	0.69 (0.50–0.94)		56%	0.65 (0.48–0.88)		1.84 (1.27–2.65)	
CK14 pattern								
Negative	59%	1.0	0.005	49%	1.0	0.003	1.0	0.002
Focal	50%	0.92 (0.65–1.28)		48%	0.83 (0.59–1.16)		2.31 (1.54–3.45)	
Diffuse	88%	0.31 (0.15–0.63)		79%	0.32 (0.17–0.63)		0.84 (0.35–2.06)	

Tumour sizes: p1, <2 cm; p2, 2–5 cm; p3, >5 cm. CI, confidence interval; CK14, cytokeratin 14; ER, oestrogen receptor; PR, progesterone receptor.

**Table 3:** Features of 'basal' versus 'non-basal' tumours

Parameter	Basal	Non-basal	Adjusted odds ratio	*p *value
Oestrogen receptor				
Positive	8 (9%)	183 (52%)		<0.0001
Negative	72 (81%)	150 (42%)	10.9 (5.08–23.2)	
Progesterone receptor				
Positive	4 (5%)	122 (33%)		<0.0001
Negative	75 (85%)	206 (58%)	11.9 (4.2–33.6)	
Axillary nodes				
Positive	37 (42%)	214 (60%)		0.0023
Negative	49 (55%)	135 (38%)	0.44 (0.27–0.72)	
Tumour size				
p1	5 (6%)	22 (6%)	1.0	0.92
p2	66 (75%)	247 (70%)	1.41 (0.50–3.97)	
p3	17 (19%)	76 (21%)	1.19 (0.38–3.7)	
Menopausal status				
Premenopausal	42 (47%)	133 (37%)	1.0	
Perimenopausal	15 (17%)	49 (14%)	1.91 (0.71–5.19)	
Postmenopausal	33 (38%)	164 (46%)	1.31 (3.80)	
Mean age in years	49.9 (SE 1.30)	53.9 (SE 0.65)		0.0065

Tumour sizes: p1, <2 cm; p2, 2–5 cm; p3, >5 cm. SE, standard error.

**Table 4:** Cox regression analysis

		Overall survival		Disease-free survival	
Prognostic factor	Level	Hazard ratio (95% CI)	*p *value	Hazard ratio (95% CI)	*p *value

Basal	CK14-positive	0.78 (0.56–1.08)	0.14	0.72 (0.52–0.99)	0.05
Age group in years	<35	1.0	0.0005	1.0	0.005
	35–49	0.86 (0.54–1.37)		0.96 (0.60–1.52)	
	50–64	0.77 (0.49–1.22)		0.80 (0.51–1.26)	
	65+	1.50 (0.91–2.46)		1.43 (0.87–2.36)	
Tumour size	p1	1.0	0.052	1.0	0.042
	p2	1.45 (0.84–2.48)		1.40 (0.83–2.36)	
	p3	1.87 (1.06–3.31)		1.92 (1.10–3.33)	
Lymph node metastasis	Positive	1.83 (1.42–2.35)	<0.0001	1.81 (1.42–2.32)	<0.0001

Tumour sizes: p1, <2 cm; p2, 2–5 cm; p3, >5 cm. CI, confidence interval; CK14, cytokeratin 14.

**Table 5:** Number and sites of metastases and time taken for metastases to develop in 'basal' versus 'non-basal' tumours

Site of metastasis	Number		5-year proportion free of metastases		Hazard ratio	*p *value
	
	Basal	Non-basal	Basal	Non-basal	(95% CI)	
All sites						
No	51	169	59%	55%	0.65 (0.48–0.88)	0.005
Yes	37	186				
Bone						
No	74	245	85%	73%	0.49 (0.28–0.86)	0.01
Yes	14	109				
Liver						
No	76	265	86%	77%	0.53 (0.29–0.97)	0.035
Yes	12	89				
Brain						
No	75	327	85%	93%	1.92 (0.27–1.01)	0.051
Yes	13	27				
Lung/pleura						
No	65	261	73%	77%	0.92 (0.58–1.46)	0.72
Yes	23	93				
Skin						
No	79	316	87%	90%	0.94 (0.45–1.95)	0.87
Yes	9	38				
Pericardial/mediastinum						
No	84	342	94%	97%	1.23 (0.39–3.83)	0.72
Yes	4	12				
Nodes (non-regional)						
No	83	293	93%	84%	0.31 (0.13–0.78)	0.008
Yes	5	61				

CI, confidence interval.
